# Pediatric ARDS biomarkers: missing the random forest for the trees

**DOI:** 10.1186/s13054-019-2396-7

**Published:** 2019-03-25

**Authors:** Nadir Yehya

**Affiliations:** Department of Anesthesiology and Critical Care Medicine, Children’s Hospital of Philadelphia and University of Pennsylvania, 6040A Wood Building, 3401 Civic Center Boulevard, Philadelphia, PA 19104 USA

Acute respiratory distress syndrome (ARDS) is characterized by acute onset of diffuse bilateral pulmonary edema and severe hypoxemia not fully explained by cardiac dysfunction [1]. Primarily defined for adults, ARDS affects 10% of mechanically ventilated children in pediatric intensive care units (PICUs) [2], with a mortality rate of 20% in modern cohorts [3, 4]. ARDS is heterogeneous, with patients having distinct co-morbidities and inciting etiologies (pneumonia, non-pulmonary sepsis). This heterogeneity has contributed to negative trial results in adults and pediatrics, as therapies effective in some patients are ineffective in others [5]. Methods to reduce heterogeneity including sub-phenotyping using protein and mRNA biomarkers have been proposed for improving patient selection for future clinical trials [6]. Biomarkers have also been proposed to predict development of, accurately diagnose, and prognosticate ARDS. Biomarkers may also provide insight into ARDS pathophysiology, which remains remarkably imprecise despite 50 years of research.

Following the lead of our adult colleagues [7], pediatric ARDS has recently experienced an explosion of manuscripts describing (primarily circulating) protein biomarkers for use in prognostication and risk stratification (reviewed in [8]). These biomarkers include damage-associated molecular patterns, inflammatory proteins (interleukins, cytokines), coagulation-associated proteins, markers of endothelial damage, and a few proteins putatively representing alveolar epithelial damage. The more promising biomarkers, such as the soluble receptor for advanced glycation end-products (sRAGE) [9], angiopoietin-2 [9, 10], and thrombomodulin [11], are strongly associated with mortality and potentially relate to pathophysiology.

However, the utility of these biomarkers expressly depends upon their intended use, which is intimately related to the epidemiology of pediatric ARDS. First, most children with ARDS do not die of hypoxemia, the defining hallmark of ARDS. Multisystem organ failure and withdrawal due to poor neurologic prognoses or underlying co-morbidities are responsible for most deaths [3]. Second, mortality is much lower in pediatric ARDS, which is why it is rarely chosen as the primary outcome in trials. Rather, composites such as ventilator-free days are more common. Unfortunately, few biomarkers have been associated with ventilator duration, so their utility in prognosticating the most common outcome used in pediatric ARDS trials is unknown. These preceding two points therefore suggest that the majority of “ARDS” biomarkers published to date are not specific for ARDS; rather, they are identifying mortality risks associated with either severe inflammation or non-specific tissue damage, with little indication that they relate to a pulmonary process like ARDS. This includes markers putatively related to alveolar epithelial damage, such as sRAGE, which is primarily expressed in lung epithelial cells. However, levels of sRAGE are *also* associated with non-pulmonary organ failures (and mortality) in pediatric ARDS [9], consistent with the expression of sRAGE in non-pulmonary tissues, including endothelial cells. It is possible that peripheral blood is the wrong compartment to identify a biomarker specific for pediatric ARDS and that investigating the proteome of the alveolar space may be more useful. However, this logical proposal confronts the reality that bronchoalveolar lavage sampling of ARDS in children is far rarer than in adults and would require extensive resources and practice change. Thus, a biomarker specific for pediatric ARDS remains elusive, and we continue to rely on clinical criteria for diagnosis and prognostication [12].

This is not to imply that these biomarkers cannot be useful. A subtype of pediatric ARDS characterized by elevated angiopoietin-2, for example, may benefit from a treatment targeting angiopoietin signaling, an example of predictive enrichment. The prognostic utility of certain biomarkers may assist with identifying a subgroup at high risk for mortality (e.g., prognostic enrichment) and thus appropriate for trials of high-risk therapies, such as high-frequency oscillatory ventilation or extracorporeal support. However, what should be clear is that the existing biomarkers are not identifying pathophysiology or risk stratification *specific to* pediatric ARDS. Angiopoietin-2, for example, has prognostic utility in pediatric sepsis, as well [13], and does not necessarily implicate ARDS-specific pathophysiology.

This, therefore, is the central question as we see more publications on biomarkers in pediatric ARDS: how do we intend to use the biomarker? If used to predict or diagnose ARDS, then the biomarker (or, more likely, panel of biomarkers) should be more specific for ARDS than what has been published to date. If used for prognostic or predictive enrichment, then the utility of the biomarker should be tested and framed appropriately, as it still may be useful, despite lacking specificity for a pulmonary process like ARDS. If used to identify pathophysiology, then studies should be clear regarding whether they are identifying pathways specific to ARDS, or to organ failures in any inflammatory syndrome.

This is an area in which we can follow the lead of our oncology colleagues. The recent successes of “tumor-agnostic” therapies, in which therapies designed around positive biomarkers (e.g., anti-programmed cell death-1 and tropomyosin receptor kinase), rather than an anatomic or histologic cancer type, are a paradigm shift [14]. Critical care is mired in the imprecise terminology of oncology 50 years past, using syndromic terms such as “sepsis” and “ARDS.” However, critical care syndromes demonstrate significant overlap of presentation, and potentially pathophysiology, which is the exact scenario in which biomarkers can play a role in more precisely defining the true pathology. We, too, can shift our paradigm, and this may point us towards the most efficient use of these biomarkers. In the near future, pediatric critical care may not be caring for children with sepsis, ARDS, traumatic brain injury, or post-cardiac arrest syndrome; rather, we may be discussing angiopoietin-dysregulated endotheliopathy, sRAGE-positive organ failures, and human-leukocyte antigen DR-deficient immunosuppression. Thus, the argument regarding how to advance the promise of precision medicine is not whether we should be better lumpers or splitters, but whether we should radically change how we view our patients.
